# King Edward VII. Sanatorium

**Published:** 1906-06-23

**Authors:** 


### KING EDWARD VII. SANATORIUM.

### OPENING BY THE KING.

The King and Queen's visit to Midhurst on the 14th instant to open the King Edward VII. Sanatorium on the Sussex hills is an occasion which may prove memorable in history as marking the extraordinary personal interest which their Majesties undoubtedly take in everything which tends to help the cure of disease, or to alleviate human suffering. Invitations were limited to about 250.

The King and Queen were attended by Lord Granard, Lady Lansdowne, Major G. Holford, and Major Fritz Ponsonby. Midhurst station was reached at 4 o'clock, where their Majesties were received by the Duke of Norfolk, Lord Lieutenant, of Sussex, Mr. Philip Secretan, Sheriff, the Under-Sheriff, the Chairman of the West Sussex County Council, and General Lord Methuen, General Officer commanding the district. A guard of honour was supplied by the second Volunteer Battalion of the Royal Sussex Regiment. Their Majesties were escorted on their journey to the sanatorium by a troop of Sussex Yeomanry, where a guard of honour was mounted by the Second Volunteer Battalion of the Royal Sussex Regiment. After the National Anthem had been sung by some hundreds of children, the King and Queen entered the Sanatorium, and were received by His Majesty's Advisory Committee, of which Sir William Broadbent is the Chairman. The entrance-hall, which was converted into a reception-room for the occasion, contains an inscription to the effect that the foundation-stone of the building was laid on November 3, 1903, by the King. On entering the dining-hall, where the addresses were read, a bouquet was offered to Her Majesty by Miss Katherine Broadbent, and graciously accepted.

#### Sir William Broadbent's Address.

The Chairman's address was as follows:—

May it please your Majesty,—The Advisory Committee honoured by your Majesty's command to supervise the construction of a sanatorium for the open-air treatment of early and curable cases of pulmonary consumption occurring in certain classes of the community, beg humbly to place before your Majesty a brief report of their proceedings, which have resulted in the erection of the group of buildings to be opened by your Majesty and the Queen to-day. With a view of eliciting new ideas and encouraging originality of design, prizes of £500, £300, and £100 were, at the suggestion of your Majesty, offered for essays on the subject, and in order that advantage might be taken of all available experience in the structural arrangements of similar institutions, the architect appointed, Mr. H. Percy Adams, was directed to visit the newest and most important of the existing sanatoria. Members of the committee also visited the chief European institutions of the kind, and it is believed that all the improvements suggested by experience are embodied in the sanatorium to be known by your Majesty's name. The class for which special provision is to be made includes those belonging to the various professions who may fall victims to phthisis and are unable to afford the expense incident to treatment in private sanatoria, clergymen and other ministers of religion, medical men, members of the teaching profession, together with clerks and others employed in business offices, or members of their families. By your Majesty's express desire the wealthy classes are not to be altogether excluded from the advantages of a sanatorium which, if it realises your Majesty's beneficent intention, is to be the best of its kind, and twelve rooms are set apart for their reception. It is unnecessary to enter into a detailed description of the building, as every point can be explained in the course of the inspection to be made by your Majesties. The main block is devoted entirely to the patients, each of whom has a separate room with a balcony, on to which the bed can be wheeled if necessary, the upper story being set back in order that the balcony for this floor should not interfere with the light and air of the rooms below. The twelve rooms for the well-to-do are in the centre, the eighty-eight for the others in the wings. Recreation-rooms are provided on the ground floor, while in the basement is a hydro-therapeutic department. The administration block, containing the dining-rooms, nurses' rooms, and kitchen, with its accessories, is separated from the main building by an ornamental garden. The communication between the two is by a corridor, permitting of the direct access of nurses and domestic servants to the grand corridor leading to the patients' rooms. Annexed to the administration block are the rooms of the resident medical staff, the consultation-rooms, and the operating-theatre, and in a convenient situation, isolated from the other parts of the sanatorium, is the pathological laboratory. A clinical and research laboratory is also provided, as it is intended that scientific work, both preventive and curative, shall be one of the prominent objects of the sanatorium. The laundry is fitted with all the latest appliances, and special precautions are taken for sterilisation of the linen. Adjoining it is the engine-house, from which is supplied the motor power for the dynamos that generate the electric light and the steam for heating the building. Your Majesty will see with particular interest the chapel erected at the expense of Sir John Brickwood. It is entirely original in plan, consisting of two naves at right angles to each other, meeting in a chancel common to both. The sides enclosing the angle which looks to the south are formed by open arches protected only by a cloister. By this admirable arrangement divine service will practically be conducted in the open air, warmth being provided by heating the floor. The site which the Advisory Committee were so fortunate as to secure is singularly beautiful and admirably fitted for all the requirements of a sanatorium. Its aspect on the southern slope of a hill, the height above the sea, the protection from the north and east afforded by the pine woods, the sandy soil, the dry air, the proportion of sunshine, the freedom from dust, the facilities for graduated walks on the rising ground, constitute a combination of advantages rarely to be met with. The Committee are confident that the natural advantages of the site, with skilled medical treatment in a perfectly appointed building, will result in the restoration to health of many of those suffering from early consumption, and in the hope and belief that in years to come blessings will be invoked on your Majesty's name for the generous thought which prompted the erection of this institution, your Committee now humbly pray that your Majesty will be pleased to declare the sanatorium open.

### The King's Reply.

His Majesty then made the following speech :—

It gives the Queen and myself great pleasure to be here to-day to open this magnificently situated building. I thank the most generous donor through whose munificence I have been enabled to found this sanatorium which bears my name, and I am well assured that it will be a great pleasure to him to know that the noble gift which he placed at my disposal has been so usefully bestowed, and that it will be the means of affording relief to those suffering from the devastating scourge of tuberculosis. I congratulate the members of the Advisory Committee upon the wholehearted manner in which they have carried out the trust I reposed in them; and I thank them for the inestimable value of their advice and experience, and for the expenditure of time and labour which, despite the many calls upon them, they have given to the work. It is my desire that this institution shall afford accommodation for that large class of persons of slender means, in professional and other employments, for which no provision for sickness of this kind at present exists. It is also my wish that those persons of larger means who can afford to pay for treatment here should not be entirely excluded from the advantages to be derived from this institution, and I have accordingly decided that a small number of beds shall be reserved for them. It has ever been my endeavour, and that of the Queen, to do all within our power to mitigate suffering and to check the ravages of disease. The Queen has shown her deep interest in the fight against tuberculosis by becoming the patron of the proposed "Queen Alexandra Sanatorium" at Davos, and by permitting that institution to be called by her name; and it is our earnest hope that the sanatorium which is now opened, and its research laboratories, equipped with every resource of modern science, may assist to advance the physiological knowledge of pulmonary diseases, and that this institution may, by treating the disease in its early stages, be the means of prolonging the lives of those whose career of honourable usefulness has been interrupted by this terrible malady. I pray that God's blessing may rest upon this building and upon all who work within it and all who come to it for aid, and that it may be a means of alleviating suffering and saving life for this and many generations to come.

After prayers by the Bishop of Chichester, and the singing or a hymn, His Majesty declared the building open. Sir William Broadbent then made the following presentations to His Majesty: Mr. H. Percy Adams, F.R.I.B.A., the architect, who presented an album of photographs of the building to the King; Mr. Charles Longley, the builder; Miss Jekyll, who has arranged the gardens; Dr. Noel Bardswell, senior medical superintendent; Dr. Basil Adams, assistant medical superintendent, who will have charge of the laboratory work; and Miss Blanche Trew, R.R.C., matron.

The King and Queen subsequently made a thorough inspection of the buildings, commencing with the kitchen, and then on through the medical officers' quarters and the grounds to the chapel, through the medical officers' garden. They then inspected the rooms set apart for the poorer class of patients—class "A"—including the recreation-room, the writing-room, and the bathroom. The accommodation for the better-class patients—class "B"—was then inspected, and then, ascending by the lift to the second floor, their Majesties were shown over the accommodation provided for the patients who are confined to their beds. Their Majesties afterwards partook of tea in the recreation-room where Mrs. Lascelles presided at the Royal table, after which Dr. Latham and Dr. Wethered, the winners of the two prize essays on the subject of the sanatorium, were presented to their Majesties in the library. Before their departure the King and Queen signed their names in the visitors' book.

